# Symbiotic Bacteria in Gills and Guts of Chinese Mitten Crab (*Eriocheir sinensis*) Differ from the Free-Living Bacteria in Water

**DOI:** 10.1371/journal.pone.0148135

**Published:** 2016-01-28

**Authors:** Meiling Zhang, Yuhong Sun, Liqiao Chen, Chunfang Cai, Fang Qiao, Zhenyu Du, Erchao Li

**Affiliations:** 1 Laboratory of Aquaculture Nutrition and Environmental Health, School of Life Sciences, East China Normal University, Shanghai 200241, China; 2 School of Biology & Basic Medical Sciences, Soochow University, Suzhou 215123, China; Zhejiang University, CHINA

## Abstract

Aquatic animals have a close relationship with water, but differences in their symbiotic bacteria and the bacterial composition in water remains unclear. Wild or domestic Chinese mitten crabs (*Eriocheir sinensis*) and the water in which they live were collected from four sampling sites in Jiangsu and Shanghai, China. Bacterial composition in water, gills or guts of *E*. *sinensis*, were compared by high-throughput sequencing using 16S rRNA genes. Analysis of >660,000 sequences indicated that bacterial diversity was higher in water than in gills or guts. Tenericutes and Proteobacteria were dominant phyla in guts, while Actinobacteria, Proteobacteria and Bacteroidetes were dominant in gills and water. Non-metric multidimensional scaling analysis indicated that microbiota from gills, guts or water clearly separated into three groups, suggesting that crabs harbor a more specific microbial community than the water in which they live. The dominant OTUs in crab gut were related to Mycoplasmataceae, which were low in abundance in gills, showing that, like mammals, crabs have body-site specific microbiota. OTUs related to *Ilumatobacter* and *Albimonas*, which are commonly present in sediment and seawater, were dominant in gills but almost absent from the sampled water. Considering *E*. *sinensis* are bottom-dwelling crustacean and they mate in saline water or seawater, behavior and life cycle of crabs may play an important role in shaping the symbiotic bacterial pattern. This study revealed the relationship between the symbiotic bacteria of Chinese mitten crab and their habitat, affording information on the assembly factors of commensal bacteria in aquatic animals.

## Introduction

Microbiota refers to the community of microorganisms harbored in a specific ecosystem. With the great effort carried out in humans and other vertebrates, it is believed that the commensal microbes are evolutionarily stable, and positively or negatively influence host health in the gut or other organs [1,2] and contribute to the development and metabolism of the host [3,4]. Thus, much research has addressed the factors influencing the assembly of the symbiotic community[5].

Compared with mammals, the principles governing the assembly of the functional symbiotic microbiota of aquatic animals are poorly understood. Similar to terrestrial mammals, host phylogeny, diet and growth state all influence the intestinal microbial composition [6,7,8]. Many questions have been raised about the influence of habitat water on the assembly of symbiotic bacteria of aquatic animals. Sullam *et al*. examined the influence of abiotic or biotic factors on the intestinal bacterial communities of fish from different taxa, trophic levels or habitats, and the results indicated that except for trophic level and host phylogeny, salinity also influenced assembly of the gut microbiota[9]. Mouchet *et al*. assessed the intestinal microbiota in 15 fish species and showed that genetic diversity and functional diversity were both significantly influenced by diet and host phylogeny, while genetic diversity was also influenced by geography [10]. A study of wild fish showed that although invasive Asian silver carp and indigenous planktivorous gizzard lived in the same river basin, their gut microbiota were different, which may be partly due to their different food sources and physiological behavior [11]. Existing studies suggest that the relationship between the symbiotic bacteria and the environment in aquatic animals is more complex than in terrestrial animals[9].

Some research has focused on the symbiotic bacteria in organs other than the gut of aquatic animals. Larsen *et al*. comparing the skin microbiota from 102 fish specimens showed that Proteobacteria was the predominant phylum in skin, followed by Firmicutes and Actinobacteria, and each fish had its own specific skin microbiotic community [12]. The bacterial epibiont community structure in the deep-sea yeti crab has been identified and the community on the pereopods is far less diverse than at other sites such as dorsal carapace, sternum, abdomen and maxillae, suggesting that both the local environment and host-derived factors influence the establishment and maintenance of microbes associated with the surfaces of aquatic animals[1]. Gills are directly exposed to water in which the animal lives, so much research has been conducted to explore the antimicrobial function and salinity acclimation of gills [13,14]. Recently, it has been found that shipworms, which are wood-eating marine bivalves, harbored endosymbiotic bacteria in their gills and these endosymbionts produce wood-degrading enzymes [15]. However, in general, information on the relationships between the microbiota in the environment and gill bacteria is very limited[16].

Chinese mitten crab (*Eriocheir sinensis*) is an economically valuable species in China, while in Europe and North America it is considered an invasive species[17]. The mating of Chinese mitten crabs occurs when the crabs reach saline water, and the larvae undergo five zoeal stages before settlement. Juvenile crabs migrate from the estuary into fresh water where they develop into adults[18].

Because of the important roles of symbiotic bacteria in host nutrition, immunity and metabolism, also the close relationship between symbiotic bacteria and habitat, characterizing the symbiotic bacterial community is one of the best ways to unveil animal behaviors[11]. Revealing the composition of the symbiotic bacteria in Chinese mitten crab and free-living bacteria in water is an important step in aquaculture. Therefore, in this study, culture-independent methods were applied to characterize the relationship among water bacteria, gill-associated bacteria and intestinal bacteria in wild or domesticated adult Chinese mitten crab.

## Materials and Methods

### Sample Collection

Samples were collected in October 2014. Sampling sites included Yangcheng Lake (Site Y; 31°25'39.58"N, 120°47'7.21"E), a culturing tank near Yangcheng Lake (Site T; 31°30'8.41"N, 120°43'39.22"E), a culturing tank in Songjiang district, Shanghai (Site S, 30°55'59.92"N, 121°12'49.29"E), and a beach on Chongming Island, Shanghai (Site C; 31°29'50.86"N, 121°55'56.02"E) (Table 1).

**Table 1:** Characteristics of sampling sites and crabs.

Site^a^	Longitude	Latitude	Temperature (°C)	Salinity (‰)	pH	DO^b^ (mg L^-1^)	Weight of crabs (g)	Characteristics of crabs
Y	31°25'39.58" N	120°47'7.21" E	21.3	0.34	8.4	10.51	143.86 ± 18.53	Semi-natural
T	31°30'8.41" N	120°43'39.22" E	20.9	0.29	8.7	14.37	203.97 ± 27.45	Domestic
S	30°55'59.92" N	121°12'49.29" E	20.9	0.3	8.2	10.47	171.81 ± 10.30	Domestic
C	31°29'50.86" N	121°55'56.02" E	19.6	14.3	7.5	10.04	32.71 ± 12.79	Wild

^a^ Y is the center of Yangcheng Lake, Jiangsu Province. T is a culturing pond near Yangcheng Lake. S is a culturing pond in Songjiang district, Shanghai.C is located at Chongming Island, Shanghai, China.

^b^DO, dissolved oxygen.

One liter of subsurface water was collected at each sampling site using a sterile container. The temperature, pH, salinity and dissolved oxygen (DO) content of the sampled water were measured using an HQ30d portable dissolved oxygen meter (HACH, USA). Large particles suspended in water samples were removed by passage through 8-μm qualitative filter paper. The filtrate was then vacuum-filtered through a 0.22-μm polycarbonate membrane (Millipore, Billerica, MA, USA). The membrane was cut into small pieces and used for bacterial genomic DNA purification.

Only male crabs were collected and they were transported to the laboratory as soon as possible. After the measurement of weight, crabs were washed thoroughly using sterile water and disinfected with 75% ethanol for 3–5 min. Crabs were dissected immediately and the whole digestive tracts were removed[19]. Four individuals were involved in each sampling site. The gut content was collected in sterile tubes. Gills were aseptically removed, washed three times with sterile water and used for bacterial genomic DNA extraction. All experiments were conducted under the Guidance of the Care and Use of Laboratory Animals in China. This research was approved by the Committee on the Ethics of Animal Experiments of East China Normal University. The sampling process in the culturing pond has been approved by the host and the field study did not involve any endangered or protected species.

### DNA Extraction

DNA extraction was performed according to a method described previously[20]. In brief, total bacterial community DNA was isolated with an E.Z.N.A. Soil DNA Kit (OMEGA, USA). DNA yield was measured in a NanoDrop spectrophotometer (Thermo Fisher Scientific, Waltham, MA, USA). DNA quality was assessed by PCR amplification of the bacterial 16S rRNA genes.

### Illumina High-throughput Sequencing of Barcoded 16S rRNA Genes

Bacterial DNA was used as the template for amplification of 16S rRNA gene V4-V5 region[21]. The primers were 515F: 5ʹ-GTGCCAGCMGCCGCGGTAA-3ʹ and 907R: 5ʹ-CCGTCAATTCCTTTRAGTTT-3ʹ. Unique eight-base barcodes were added to each primer to distinguish the PCR products. The PCR reaction mixture (25 μL) included 0.25 U Platinum® *Pfx* DNA polymerase (Invitrogen, Carlsbad, CA, USA), 2.5 μL corresponding 10× *Pfx* amplification buffer, 0.5 mM MgSO_4_, 0.25 mM deoxynucleoside triphosphates, 6.25 pmol each primer, and 20 ng extracted DNA. The PCR program began with a 3 min denaturation step at 94°C; this was followed by 20 cycles of 1 min at 94°C (denaturation), a 1 min annealing step (65°C to 57°C with a 1°C reduction every two cycles followed by one cycle at 56°C and one cycle at 55°C), and a 1 min elongation step at 72°C; then a final 6 min extension at 72°C. The PCR products were purified using an AxyPrep^TM^ DNA Gel Extraction Kit (Axygen, Hangzhou, China). Thirty nanograms of each purified PCR pr oduct were subjected to Illumina-based high-throughput sequencing (Majorbio Bio-Pharm Technology Co., Ltd., Shanghai, China). The sequences obtained in this paper are available in the GenBank Sequence Read Archive database with BioProject number PRJNA 285008

### Bioinformatics and Statistical Analyses

Raw fastq files were demultiplexed and quality-filtered using QIIME (version 1.17) [22]. Reads containing more than two mismatches to the primers or more than one mismatch to the barcode were discarded and reads of <50 bp were removed. Reads of 250 bp were truncated at any site receiving an average quality score of <20 over a 50 bp sliding window.

Operational Taxonomic Units (OTUs) were clustered with 97% similarity cutoff using UPARSE (version 7.1; http://drive5.com/uparse/) [23] and chimeric sequences were identified and removed using UCHIME[24]. The phylogenetic affiliation of each 16S rRNA gene sequence was analyzed by the RDP Classifier (http://rdp.cme.msu.edu/) against the SILVA database using a confidence threshold of 70%.

Rarefaction curves were created in Mothur to determine whether sequencing depth was sufficient to cover the expected number of OTUs at the level of 97% sequence similarity. Taxonomic richness and diversity estimators were determined for each library in Mothur. All these indices were estimated based on OTU abundance matrices. ACE and Chao were used to reflect community richness[25,26]. Diversity was assessed using Shannon indices[27].The mean of the estimated parameters was used for comparisons between samples. For similarity measurement among the bacterial communities in the samples, the Bray–Curtis similarity index was used to compare samples according to the abundance of OTUs in samples. Non-metric multidimensional scaling (NMDS) based on weighted UniFrac distance was used to visualize the pairwise Unifrac distance among samples. Heatmap was drawn based on the OTUs in all samples from water, gills or guts. Network analysis was conducted using the igraph package in R to visualize the distribution of major bacterial groups (average abundance >0.01%) in water, gill or gut samples.

## Results

### Sampling Sites and General Information on the Crabs

Four sampling sites were used in this study. Two of them were located in Jiangsu Province, China and the other two were in Shanghai, China. The temperature varied from 19.6–21.3°C (Table 1). Site C is located near the East China Sea, so the water salinity is higher than other sampling sites. Water at site C has a lower pH, and water at site T, which is a culturing pond near Yangcheng Lake, has higher dissolved oxygen content than other sites.

Crabs from sites T and S were domesticated in ponds, which were around 5,000 m^2^, and crabs from site Y were semi-natural because they were cultured in Yangcheng Lake, which is around 120 km^2^. Crabs from site C were wild. The average weight of crabs at sites Y, T, S and C were shown in Table 1.

### General Analyses of High-throughput Sequencing

After filtering out low-quality reads, 8521 to 10103 reads were collected at the various sites for water sample analyses, 9961 to 15594 reads were collected for gill-associated bacterial composition analyses, and 9421 to 14147 reads were obtained for intestinal bacterial community analyses. Because bacterial-specific primers were used, all sequences found in this study were assigned as bacteria. Rarefaction analysis indicated that sufficient sampling depth was achieved for each sample (S1 Fig). To estimate and compare the bacterial diversity in each group, bacterial richness and diversity indices were calculated from the proportion of OTUs. The microbial complexities in water, gills and guts of crabs, were estimated based on alpha-diversity indices (Chao1 and Shannon indices). Bacterial community in water showed higher alpha-diversity indices than those from gills or guts (S2 Fig).

### Microbial Community Compositions

**Fig 1:**
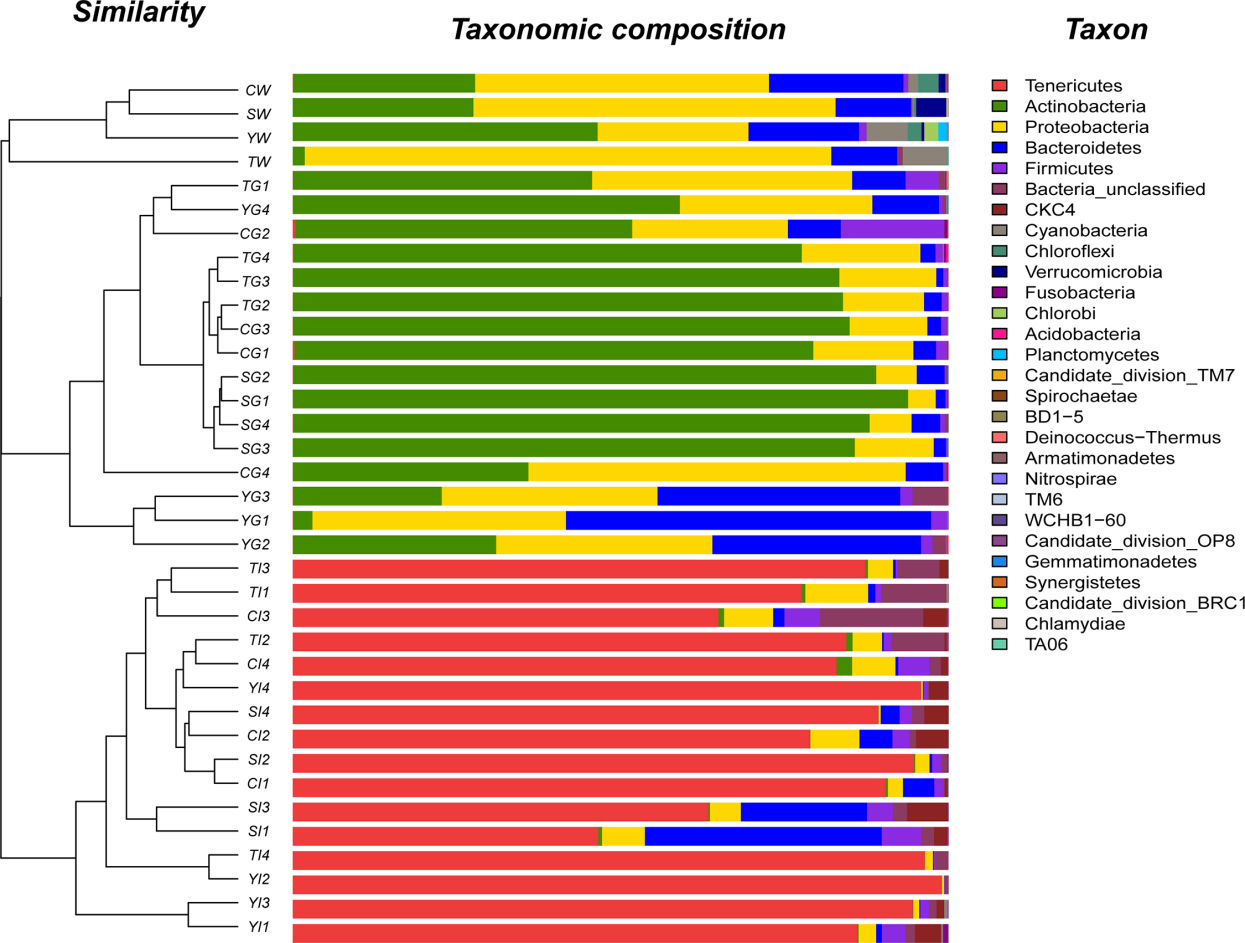
Frequency distribution of bacterial phyla in water, gills and guts. Similarity among samples was calculated using the Bray-Curtis method.

The phylogenetic classification of sequences from water samples identified 17 different phyla or groups (Fig 1). Proteobacteria (22.98% to 80.28%), Bacteroidetes (10.06% to 20.47%), Actinobacteria (1.83% to 46.50%) and Cyanobacteria (0.30% to 6.70%) were the dominant phyla in water and these four groups accounted for 92.60% to 98.87% of the total reads.

The gill-associated bacteria were dominated by Actinobacteria (2.90% to 93.82%), Proteobacteria (4.21% to 57.52%), Bacteroidetes (1.06% to 55.67%) and Firmicutes (0.21% to 15.69%). These four phyla accounted for 94.40% to 99.86% of the total reads.

The intestinal bacteria were dominated by five phyla, including Tenericutes (46.62% to 98.94%), Bacteroidetes (0.08% to 36.11%), Proteobacteria (0.23% to 5.44%), Firmicutes (0.21% to 6.01%) and CKC4 (0.03% to 6.25%). These five groups represented 89.23% to 99.75% of the total reads.

### Microbial Community Similarity Among Water, Gills and Gut Bacteria

**Fig 2:**
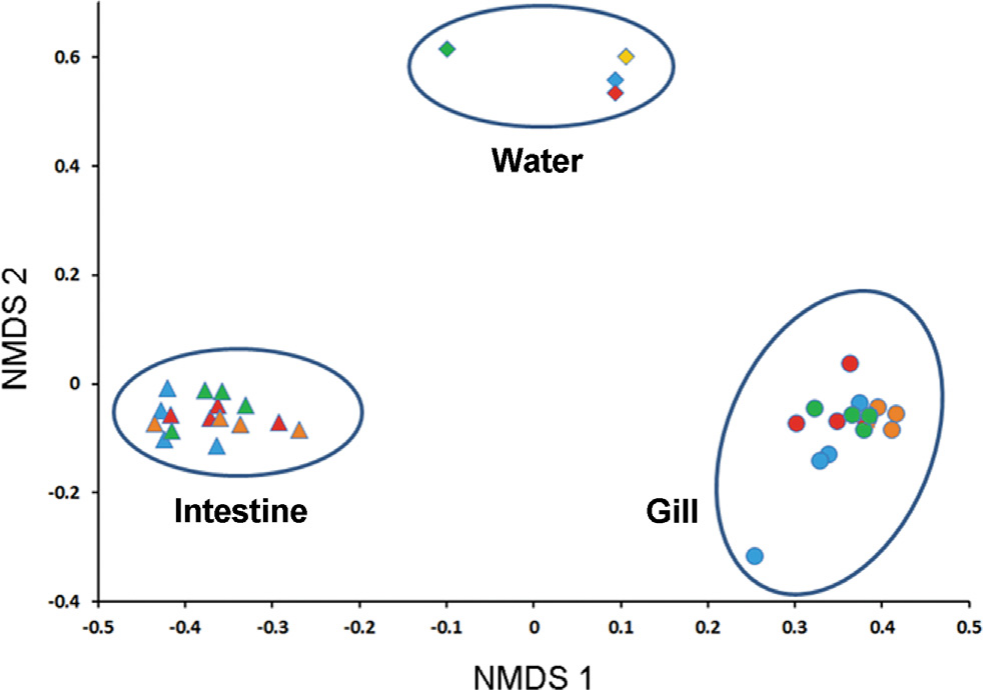
NMDS plot showing microbial community differences in water, gills and guts. The distances were determined using the Bray-Curtis method with relative abundance of OTUs. Green represents samples from site T, yellow samples from site S, blue samples from Y, and red samples from C. Diamonds indicate samples from water, triangles samples from gut, and circles samples from gills.

A NMDS plot was used to compare the similarity in bacterial compositions in water, gills and guts from the crabs, taken from four sampling sites. Bacteria in water, gills and guts separated from each other in this analysis (Fig 2). Bacterial communities from gills and guts formed distinct clusters. The clustering pattern among samples was not influenced by the sampling location.

**Fig 3:**
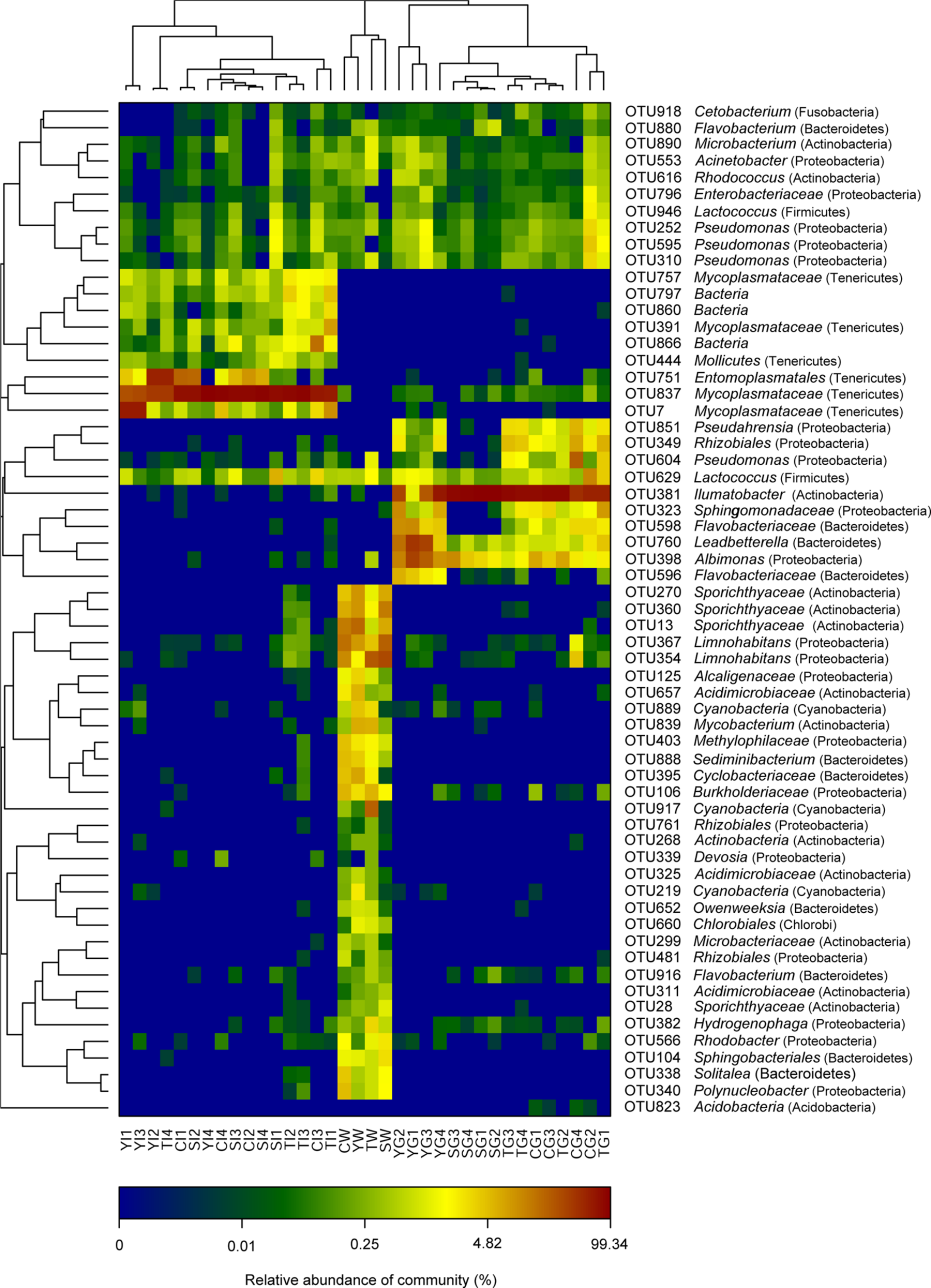
Heatmap analysis of the OTUs in all samples. The color of the bar represents the abundance of each OTU in a given sample. The affiliation of each OTU is indicated on the right.

The abundance and phylogenetic affiliations of OTUs found in water, gill or gut samples were studied by heatmap analysis (Fig 3). Thirty-five OTUs were detected in water samples. Among them, OTU13, OTU270 and OTU338 (Sporichthyaceae), OTU125 (Alcaligenaceae), OTU340 (*Polynucleobacter*), OTU395 (Cyclobacteriaceae), OTU403 (Methylophilales), OTU839 (Mycobacteriaceae), and OTU888 (*Chitinophagaceae*) were found in water but were absent from gill samples. Numerous other OTUs were more dominant in water than in other samples, including OTU106 (*Polynucleobacter*), OTU354 and OTU367 (*Limnohabitans*), OTU360 (Sporichthyaceae), and OTU657 (*Acidimicrobiaceae*).

Sixteen OTUs were detected in most (>80%) gill samples. Among them, OTU381 (*Ilumatobacter*), OTU398 (*Albimonas*) and OTU760 (*Leadbetterella*) were dominant, and the proportion of these OTUs varied from 1.33% to 93.6%, 0.96% to 33.15% and 0.16% to 39.56%, respectively, in samples from the four sites. Interestingly, these OTUs were almost absent from water.

Sixteen OTUs were detected in most (>80%) gut samples. Among these, OTU837, OTU7, OTU391, and OTU757 (all affiliated to the *Mycoplasmataceae*) were dominant, and their proportions varied from 23.9% to 94.76%, 0.05% to 53.36%, 0.01% to 6.32% and 0.07% to 2.27% in samples from the four sites, respectively. The proportion of these four OTUs in gut samples varied from 35.78% to 95.10%, while they had low abundance in gills and were almost absent from water.

**Fig 4:**
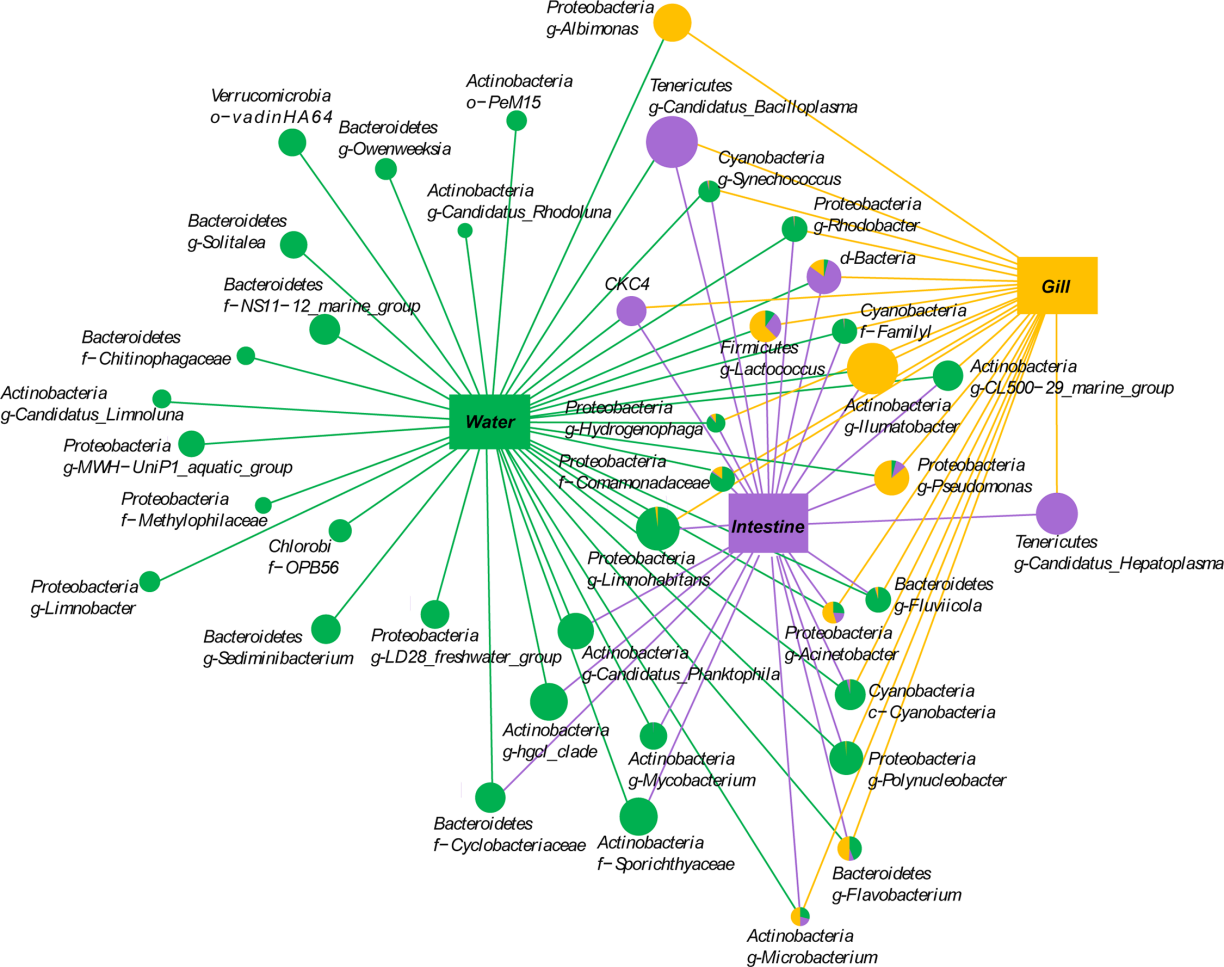
Network analysis visualizing the unique and shared bacterial groups in guts and gills of Chinese mitten crab and in the water in which they live. Node sizes correspond to the mean relative abundance of each bacterial group. The proportion of shared bacteria is represented by the different colors in the pie charts; if the proportion of the bacterial group is <0.1% in gill, gut or water, these bacterial groups are shown with a line only.

To investigate the microbial communities in different samples, the distribution of the major bacterial groups was visualized by network analysis (Fig 4). Many bacterial groups were present in gut, gill and water, but the abundance of these bacterial groups varied. Water has a more diverse microbial community while gills and guts harbor their own specific microbiota. For instance, *Ilumatobacter*, *Albimonas* and *Pseudomonas* were more dominant in gill, while *Candidatus Bacilloplasma*, *Candidatus*, *Hepatoplasma* and CKC4 were more dominant in gut.

## Discussion

Aquatic animals have a persistent, close relationship with the water in which they live. Some studies have tried to identify the influencing factors or origin of the intestinal bacteria of aquatic animals[9,20]. However, patterns in the bacterial composition in water and host-associated organs are inconsistent. The intestinal bacterial communities in black tiger shrimp (*Penaeus monodon*) varied significantly between different commercial farms and were distinct from their rearing water [28]. Our previous research also compared the bacterial composition between the shrimp (*Litopenaeus vannamei*) gut and the culturing water, and the results indicated that the dominant bacteria in water were *Cyanobacteria* while the dominant members in shrimp gut were Proteobacteria and Tenericutes[20]. Wu *et al*. compared the composition, diversity and origin of the bacterial community in grass carp gut and revealed that the bacterial community in the gut is similar to those from the culture water and sediment[29]. Information about the bacterial composition in the gills, which are directly exposed to water, was very limited. A recent study revealed the microbiota at the organ scale in Manila clam (*Ruditapes philippinarum*) and indicated that Manila clam microbial structure differed with organs, but the bacterial composition in each organ was not shown[16]. In the present study, the bacterial compositions in habitat water, gut or gills of Chinese mitten crabs collected from four sites were identified, and our results suggest that water, gills and guts have their own bacterial patterns, although some bacterial groups were shared by all samples.

The bacterial diversity in the water in which the crabs live is higher than the diversity in gills or guts of Chinese mitten crab, which is consistent with a previous study in fish[7], suggesting that aquatic animals specialize their symbiotic flora relative to the environment in which they live. Similar to previous research, Proteobacteria, Actinobacteria, Bacteroidetes and Cyanobacteria were the dominant phyla in water in the present study. Because gills are in constant contact with bacteria present in water, these phyla (except Cyanobacteria) were also dominant in gills, but the presence of particular OTUs differed between water and gills, such as OTUs related to *Limnohabitans* and Sporichthyaceae were dominant in water but almost absent from the gill and gut samples. Sporichthyaceae have previously been found to be dominant in water [30] and *Limnohabitans* are widely distributed in freshwater habitats but prefer nonhumic habitats[31]. Considering that Chinese mitten crab are bottom-dwelling crustacea and *Ilumatobacter* are dominant in sediment or seashore sand, it is not surprising that *Ilumatobacter* are dominant in gills of the Chinese mitten crab[32]. *Albimonas*, which was often detected in the seawater[33], was present in gills of crabs but almost absent from water. *E*. *sinensis* mate in saline water or seawater and eggs are hatched in water with salinity of 20‰. Mitten crab larvae are euryhaline, but they still require at least 16–17‰ salinity. Mitten crab megalopaes use tidal currents to move into river systems from estuaries[18]. Given the life cycle of *E*. *sinensis*, it is possible that *Albimonas* colonized the gills of crabs when they hatched in the sea. Chen *et al*. found that *Marinifilum fragile* (Bacteroidetes), which is a marine bacterium, is present in the gut of Chinese mitten crab and they speculated that *M*. *fragile*-like bacteria originated from the sea[19]. These findings suggest that colonization by symbiotic bacteria is closely related to the behavior or life cycle of the animals concerned. The Mycoplasmataceae, which belong to the Tenericutes, are one of the most dominant families in the gut. In previous work, Mycoplasmataceae were barely detected in water or gills, and this finding suggests that the crab gut is colonized by its own particular intestinal bacterial community [19]. Chen *et al*. found that Mycoplasmataceae were dominant in the gut of Chinese mitten crab but they did not detect Mycoplasmataceae in guts of crabs from the Yangtze River estuary and Chongming Islands[19]. Our results indicate that Mycoplasmataceae were dominant in the guts of wild (samples from Chongming Island), semi-natural (samples from Yangcheng Lake) or domesticated (samples from two tanks) crabs, but their function remains unknown.

It has been found that *Vibrio* is the dominant symbiotic genus in squid. Proteobacteria and Firmicutes are dominant in the gut of fruit fly. In fish, Proteobacteria and Fusobacteria were thought to be the predominant phyla in gut. In mice gut, Firmicutes, Bacteroidetes and Proteobacteria were the most dominant members, and in human gut, Firmicutes, Bacteroidetes and Actinobacteria were the predominant phyla[34]. In the present study, we found that Tenericutes and Proteobacteria were the most dominant phyla in the gut of crab, and our previous study found that these two phyla were dominant in the gut of *L*. *vannamei*[20]. These findings suggest that there may be some evolutionary relationship between the animals and their symbiotic microbes, although functions of many symbiotic bacteria remain to be elucidated.

In conclusion, gills and guts of wild or domestic Chinese mitten crabs and the water where they live have distinct microbiota. The bacterial diversity (Chao1 and Shannon indices) in water is higher than those in guts or gills, suggesting that the host animal harbors more specific microbial flora than its environment. This study showed that the symbiotic microbiota were body site-specific within the bodies of crabs and further research to study the function(s) of the dominant bacteria in each tissue may further provide understanding of the relationship between the symbiotic bacteria and the host.

## Supporting Information

S1 Fig Rarefaction analysis of microbiota from water (a), gills (b) and guts (c). Operational taxonomic units (OTUs) were classified based on 97% sequence similarity. The rarefaction curves for all samples reached the near plateau phase, suggesting good sampling depth.
(DOCX)

S2 Fig Bacterial diversity richness(OTUs), diversity index (Shannon) and estimated OTU richness (ACE, Chao1) for bacterial diversity from water, gills and intestine. (DOCX)
