# Supplementary material for: Symbiotic Bacteria in Gills and Guts of Chinese Mitten Crab (Eriocheir sinensis) Differ from the Free-Living Bacteria in Water
Source: PLoS One. 2016 Jan 28;11(1):e0148135. doi: 10.1371/journal.pone.0148135 (PMC4731060; doi:10.1371/journal.pone.0148135)
Supplement: S1 Fig — Operational taxonomic units (OTUs) were classified based on 97% sequence similarity. The rarefaction curves for all samples reached the near plateau phase, suggesting good sampling depth. (DOCX) [file pone.0148135.s001.docx]

**S1 Fig. Rarefaction analysis of microbiota from water (a), gills (b) and guts (c)**.Operational taxonomic units (OTUs) were classified based on 97% sequence similarity. The rarefaction curves for all samples reached the near plateau phase, suggesting good sampling depth.
