# Supplementary material for: Symbiotic Bacteria in Gills and Guts of Chinese Mitten Crab (Eriocheir sinensis) Differ from the Free-Living Bacteria in Water
Source: PLoS One. 2016 Jan 28;11(1):e0148135. doi: 10.1371/journal.pone.0148135 (PMC4731060; doi:10.1371/journal.pone.0148135)
Supplement: S2 Fig — (DOCX) [file pone.0148135.s002.docx]

**S2 Fig Bacterial diversity richness(OTUs), diversity index (Shannon) and estimated OTU richness (ACE, Chao1) for bacterial diversity from water, gills and intestine.**
